# Intra-Abdominal Venous Pressure During Laparoscopic Cholecystectomy

**DOI:** 10.1155/1994/13689

**Published:** 1994

**Authors:** Kazuhiro Iwase, Tetsuto Takao, Hirotoshi Watanabe, Yasuhiro Tanaka, Tetsuo Kido, Noritusugu Ogawa, Norio Ono, Hiroshi Yoshitake

**Affiliations:** The Department of Surgery and The Department of Anesthesiology Osaka Prefectural Hospital 3-1-56, Mandaihigashi Sumiyoshi-ku, Osaka-city Osaka 558 Japan

## Abstract

Superior vena cava (SVC) and inferior vena cava (IVC) pressures were measured serially during laparoscopic cholecystectomy in which the intra-abdominal pressure was maintained at 12mmHg. The influences of alteration of position from 15 degrees head-down to 15 degrees head-up and of the operative procedure of holding the gallbladder up to the right subphrenic space on SVC and IVC pressures were mild. IVC pressure was maintained almost equal to the intra-abdominal pressure during prolonged continuous pneumoperitoneum lasting longer than 60min, while SVC pressure did not change significantly during operation. The discrepancy between SVC and IVC pressures underwent no change during continuous pneumoperitoneum.


*HPB Surgery*, 1994, Vol. 8, pp. 13–17
Reprints available directly from the publisher
Photocopying permitted by license only

© 1994 Harwood Academic Publishers GmbH
Printed in Malaysia

### Intra-Abdominal Venous Pressure During Laparoscopic Cholecystectomy

KAZUHIRO IWASE, TETSUTO TAKAO, HIROTOSHI WATANABE, YASUHIRO TANAKA, TETSUO KIDO, NORITUSUGU OGAWA, NORIO ONO, and HIROSHI YOSHITAKE*

The Department of Surgery and The Department of Anesthesiology*, Osaka Prefectural Hospital, 3-1-56, Mandaihigashi, Sumiyoshi-ku, Osaka-city, Osaka, 558, Japan

Superior vena cava (SVC) and inferior vena cava (IVC) pressures were measured serially during laparoscopic cholecystectomy in which the intra-abdominal pressure was maintained at 12 mmHg. The influences of alteration of position from 15 degrees head-down to 15 degrees head-up and of the operative procedure of holding the gallbladder up to the right subphrenic space on SVC and IVC pressures were mild. IVC pressure was maintained almost equal to the intra-abdominal pressure during prolonged continuous pneumoperitoneum lasting longer than 60 min, while SVC pressure did not change significantly during operation. The discrepancy between SVC and IVC pressures underwent no change during continuous pneumoperitoneum.

KEY WORDS: laparoscopic cholecystectomy pneumoperitoneum inferior vena cava pressure intra-abdominal pressure

### INTRODUCTION

Various experimental studies have previously shown that critical changes in cardiovascular response or intra-abdominal visceral blood flow may be induced by high intra-abdominal pressure, exceeding 20 or 40 mmHg, but that these changes are mild when the intra-abdominal pressure is lower than 20 mmHg[1,2,3]. Laparoscopic cholecystectomy with pneumoperitoneum under general anesthesia has been accepted as a procedure safe with regard to systemic hemo-dynamics if intra-abdominal pressure is maintained below 15 mmHg. Recent studies of ours, however, have demonstrated that prolonged continuous pneumoperitoneum may result in transient decreases in effective renal plasma flow and urine output even if intra-abdominal pressure is maintained at 12 mmHg during laparoscopic cholecystectomy[4,5]. We suspect that these changes in renal hemodynamics are due to the elevated pressure in the inferior vena cava and renal vein associated with elevated intra-abdominal pressure. However, no previous study has examined in detail the serial changes in intra-abdominal venous pressure which occur during laparoscopic cholecystectomy in the clinical setting when intra-abdominal pressure is less than 15 mmHg. The purpose of this study is to determine the influences of prolonged continuous pneumoperitoneum, intra-operative head-up or head-down position and operative procedures on pressure in the inferior vena cava during surgery.

### MATERIALS AND METHODS

Ten patients who underwent laparoscopic cholecystectomy (LAP) and six patients who underwent open-laparotomy cholecystectomy (OPEN) during the period June 1992 through October 1992 were studied. Patients with choledocholithiasis, liver cirrhosis chronic obstructive lung disease, or congenital or acquired heart disease were excluded from participation in this study. Preoperative laboratory data such as platelet count, hepaplastin test, prothrombin time and the plasma levels of fibrinogen and fibrinogen degradation products were normal in all patients. General anaesthesia was maintained using isoflurane and nitrous oxide following rapid induction. The intra-abdominal pressure during LAP was maintained at

12 mmHg with an electric $CO_2$ insufflator (OLYMPUS OPTICAL Co., Ltd., Tokyo, Japan). Four disposable trocars (Two 10 mm Surgiports and two 5 mm Surgiports, United States Surgical Corporation, Norwalk, USA) were placed with the patient horizontal and operative procedures were performed in the 15 degrees head-up position for patients in the LAP group. Operative procedures were performed in horizontal position for patients in the OPEN group. Attemps were made to perform intra-operative cholangiography for patients in each of the groups. No anti-thrombotic therapy was made in any patient in any group.

Following completion of induction of anesthesia, 16-gauge catheters were inserted into the intrathoracic superior vena cava (SVC) via the right internal jugular vein, and into the intra-abdominal inferior vena cava (IVC) above the renal vein and below the liver via the right femoral vein. Heart rate (HR), systemic blood pressure (BP) and SVC and IVC pressures were measured at six time points during operation, i.e., before and after initiation of pneumoperitoneum or laparotomy, 30 and 60 minutes after initiation of pneumoperitoneum or laparotomy, and before and 15 min after depneumoperitoneum or closure of the peritoneum. SVC and IVC pressures were measured 15 seconds after disconnecting the orotracheal tube from the mechanical ventilator. SVC and IVC pressures were also measured in horizontal, 15 degrees head-up and 15 degrees head-down positions before and after initiation of pneumoperitoneum or laparotomy. In six of the 10 patients in the LAP group, changes in SVC and IVC pressures were determined with the gallbladder held up to the right subphrenic space using a grasper. In three of the 10 patients in the LAP group, correlation between intra-abdominal pressure and SVC or IVC pressure was determined for the period between 60 min after initiation of pneumoperitoneum and depneumoperitoneum, when intra-abdominal pressure underwent gradual change.

Data are expressed as mean ± standard deviation. Statistical analysis was performed using the generalized Wilcoxon test or ANOVA for repeated measurements and multiple comparison (with Scheffe-Tukey). Obtained $p$ values less than 0.05 were taken to indicate statistically significant differences.

### RESULTS

### 1. Intra-Operative Water Balance and Postoperative Course

Operating time was 132 ± 28 min in the LAP group and 105 ± 39 min in the OPEN group. Intra-operative infusion rate was 0.38 ± 0.09 ml/kg/min in the LAP group and 0.41 ± 0.07 ml/kg/min in the OPEN group. Intra-operative rate of urine output was 0.07 ± 0.02 ml/kg/min in the LAP group and 0.09 ± 0.04 ml/kg/min in the OPEN group. There was no significant difference in intra-operative rate of infusion or rate of urine output between the two groups. Between 5 to 10 mg frusemide was administered during operation to four of the 10 patients in the LAP group.

Postoperative course was uneventful in all patients in the OPEN and LAP groups. Postoperative abnor-

**Table 1** Serial changes in HR, BP and SVC and IVC pressures.

| | | *Time points* 1 | 2 | 3 | 4 | 5 | 6 |
|---|---|---|---|---|---|---|---|
| LAP group | HR (beats/min) | 80 ± 9 | 79 ± 9 | 82 ± 10 | 83 ± 9 | 81 ± 11 | 76 ± 11 |
| | Systolic BP (mmHg) | 127 ± 24 | 152 ± 25 | 136 ± 18 | 139 ± 23 | 142 ± 21 | 132 ± 21 |
| | Diastolic BP (mmHg) | 66 ± 16 | 86 ± 18 | 77 ± 14 | 80 ± 14 | 82 ± 17 | 78 ± 19 |
| | SVC pressure (mmHg) | 3.2 ± 1.2 | 3.6 ± 1.0 | 3.8 ± 1.5 | 2.9 ± 1.2 | 4.0 ± 1.1 | 3.9 ± 1.4 |
| | IVC pressure (mmHg) | 3.7 ± 1.6 | 11.8 ± 2.0 *#†ρ | 10.5 ± 1.9 *#†ρ | 9.7 ± 1.6 *#†ρ | 10.5 ± 2.2 *#†ρ | 4.4 ± 2.3 |
| OPEN group | HR (beats/min) | 79 ± 14 | 78 ± 13 | 83 ± 14 | 78 ± 11 | 77 ± 11 | 78 ± 12 |
| | Systolic BP (mmHg) | 122 ± 13 | 150 ± 24 | 141 ± 26 | 132 ± 20 | 128 ± 21 | 136 ± 19 |
| | Diastolic BP (mmHg) | 68 ± 14 | 88 ± 22 | 80 ± 21 | 78 ± 22 | 79 ± 19 | 76 ± 20 |
| | SVC pressure (mmHg) | 3.4 ± 1.8 | 3.9 ± 1.6 | 4.1 ± 1.9 | 3.3 ± 2.0 | 3.9 ± 1.4 | 3.8 ± 2.0 |
| | IVC pressure (mmHg) | 3.8 ± 2.1 | 4.8 ± 2.4 | 4.9 ± 2.0 | 3.9 ± 2.2 | 4.4 ± 1.9 | 4.5 ± 2.1 |

Data are expressed as mean ± standard deviation. *: $p < 0.05$ v.s. time point "1". #: $p < 0.05$ v.s. time point "6". †: $p < 0.05$ v.s. OPEN group. $\rho$: $p < 0.05$ v.s. SVC pressure. 1: following initiation of anesthesia. 2: following initiation of pneumoperitoneum or laparotomy. 3: 30 min after initiation of pneumoperitoneum or laparotomy. 4: 60 min after initiation of pneumoperitoneum or laparotomy. 5: preceding depneumoperitoneum or closure of the peritoneum. 6: following depneumoperitoneum or closure of the peritoneum.

malities in blood coagulation was not recognized in any patient in any group.

### 2. Serial Changes in HR, BP and SVC and IVC Pressures (Table 1, Figure 1)

There were no significant changes in HR, BP or SVC pressure during operation in either the LAP or the OPEN groups. There were no significant differences between the LAP and the OPEN groups in HR, BP or SVC pressure at any time point tested. There was no significant change over time during operation in IVC pressure in the OPEN group. IVC pressures at four time points tested during pneumoperitoneum were all significantly higher than those prior to initiation of pneumoperitoneum and following depneumoperitoneum in the LAP group. There was no significant change in IVC pressure during pneumoperitoneum in the LAP group. There were no significant differences in IVC pressures between the LAP and the OPEN groups

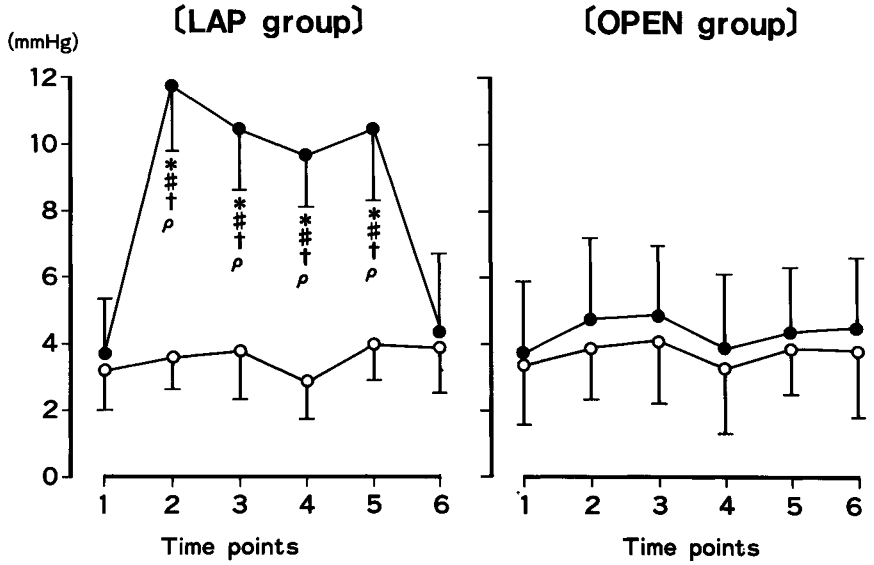

**Figure 1** Serial changes in SVC and IVC pressures. Open circles represent SVC pressures. Closed circles represent IVC pressures. Data are expressed as mean + standard deviation. *:$p < 0.05$ v.s. time point "1". #; $p < 0.05$ v.s. time point "6". †: $p < 0.05$ v.s. OPEN group: $p < 0.05$ v.s. SVC pressure. 1: following initiation of anesthesia. 2: following initiation of pneumoperitoneum or laparotomy. 3: 30 min after initiation of pneumoperitoneum or laparotomy. 4: 60 min after initiation of pneumoperitoneum or laparotomy. 5: preceding depneumoperitoneum or closure of the peritoneum. 6: following depneumoperitoneum or closure of the peritoneum.

**Table 2** Influence of position on SVC and IVC pressures.

| | *Time points* | *Position* | *SVC pressure* (mmHg) | *IVC pressure* (mmHg) |
|---|---|---|---|---|
| LAP group | before initiation of pneumoperitoneum | 15° head-up | 3.3 ± 1.3 | 3.9 ± 1.9 |
| | | horizontal | 3.2 ± 1.2 | 3.7 ± 1.6 |
| | | 15° head-down | 3.3 ± 1.1 | 3.6 ± 1.7 |
| | after initiation of pneumoperitoneum | 15° head-up | 3.7 ± 1.2 | 12.2 ± 1.8 |
| | | horizontal | 3.6 ± 1.0 | 11.8 ± 2.0 |
| | | 15° head-down | 3.5 ± 1.4 | 10.4 ± 2.4 |
| OPEN group | before laparotomy | 15° head-up | 3.5 ± 1.4 | 3.6 ± 1.9 |
| | | horizontal | 3.4 ± 1.8 | 3.8 ± 2.1 |
| | | 15° head-down | 3.6 ± 1.9 | 3.9 ± 2.0 |
| | after laparotomy | 15° head-up | 4.1 ± 2.0 | 4.0 ± 1.8 |
| | | horizontal | 3.9 ± 1.6 | 4.8 ± 2.4 |
| | | 15° head-down | 4.2 ± 1.8 | 4.4 ± 1.9 |

Data are expressed as mean ± standard deviation.

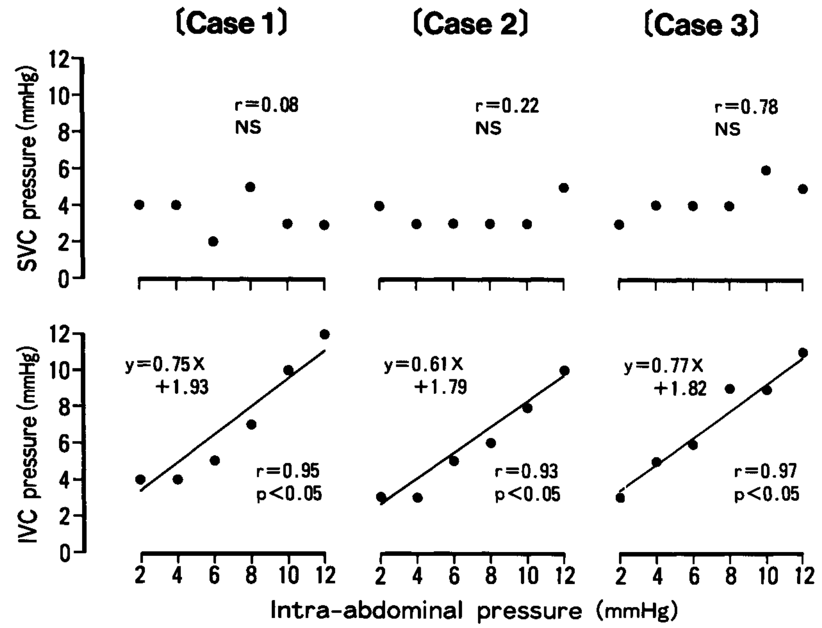

**Figure 2** Correlation between intra-abdominal pressure and SVC and IVC pressures.

at the time points tested prior to initiation of pneumoperitoneum or laparotomy and after depneumoperitoneum or closure of the peritoneum. IVC pressures at four time points during pneumoperi-toneum in the LAP group were significantly higher than those during laparotomy in the OPEN group.

### 3. Influence of Position or Holding up of the Gallbladder on SVC and IVC Pressures

During neither pneumoperitoneum or laparotomy did patient position have any effect on SVC or IVC pressure (Table 2). In the LAP group, SVC pressure was $3.7 \pm 1.4$ mmHg and IVC pressure was $11.2 \pm 3.4$ mmHg without holding up of the gallbladder, while SVC pressure was $3.8 \pm 1.5$ mmHg and IVC pressure was $11.6 \pm 3.8$ mmHg with holding up of the gallbladder. Whether the gallbladder was held up or not during pneumoperitoneum had no effect on either SVC or IVC pressure.

### 4. Correlation Between Intra-Abdominal Pressure and SVC or IVC Pressure (Figure 2)

SVC pressure was unaffected by raising the intra-abdominal pressure from 2 to 12 mmHg. However, the elevation of intra-abdominal pressure was associated with gradual elevation of IVC pressure.

### DISCUSSION

It has been considered that IVC pressure is elevated due to direct pressure when the intra-abdominal pressure is elevated[2,6]. It has been reported that cardiac output fell following a decrease in venous return from the intra-abdominal viscera and the lower extremities in association with an intra-abdominal pressure above 15 or 20 mmHg[6,7]. Results of another study suggested that cardiac output was increased following the elevation of SVC pressure by Starling's law when the intra-abdominal pressure was less than 25 mmHg[8]. It has been though that this elevation of SVC pressure is the direct result of pressure by the diaphragm[8,9]. However, previous studies of ours have shown that heart rate, systemic and pulmonary blood pressures, central venous pressure, pulmonary arterial wedge pressure and cardiac output do not change significantly during laparoscopic cholecystectomy performed in a clinical setting when the intra-abdominal pressure is maintained at 12 mmHg[4]. In the present study, indeed, the influence of elevated intra-abdominal pressure on SVC pressure was found to be mild when intra-abdominal pressure was less than 12 mmHg. We believe that sufficient quantity of infusion under general anesthesia may play a role in maintaining this stability of SVC pressure and cardiac output in the clinical setting.

The exact nature of serial change in IVC pressure during prolonged continuous pneumoperitoneum with constant SVC pressure and cardiac output in the clinical setting remains unclear. The present study demonstrated that IVC pressure continued to reflect the intra-abdominal pressure during pneumoperitoneum, and that the discrepancy between SVC and IVC pressures did not change during pneumoperitoneum. The operative procedure of holding the gallbladder up to the right subphrenic space in the 15 degrees head-up position has occasionally been used for laparoscopic cholecystectomy[10]. It has been reported that significant changes in hemodynamic parameters occur in the 25 degrees head-down position[8]. It is possible that holding up of the gallbladder, and consequently of the liver, may affect the shape of the sectional plane of the compressed IVC. In the present study, the influences of operative procedure and of variation of position on IVC pressure were found to be mild.

In conclusion, IVC pressure was maintained almost equal to the intra-abdominal pressure during prolonged continuous pneumoperitoneum in spite of variation in operative procedures and traditionally-used alterations of position during laparoscopic cholecystectomy. The clinical message from our findings is that much attention should be paid to the continuous discrepancy between SVC and IVC pressures when operating time is prolonged.

### REFERENCES

1. Kashtan, J., Green, J. F. and Parsons, E. Q. (1981) Hemodynamic effects of increased abdominal pressure. *J. Surg. Res.*, **30**, 249–259.
2. Barnes, G. E., Laine, G. A., Giam, P. Y., Smith, E. E. and Granger, H. J. (1985) Cardiovascular responses to elevation of intra-abdominal hydrostatic pressure. *Am. J. Physiol.*, **248**, 208–213.
3. Caldwell, C. B. and Ricotta, J. J. (1987) Changes in visceral blood flow with elevated intra-abdominal pressure. *J. Surg. Res.*, **43**, 14–20.
4. Iwase, K., Takenaka, H., Ishizaka, T., Ohata, T., Oshima, S. and Sakaguchi, K. (1993) Serial changes in renal function during laparoscopic cholecystectomy. *Eur. Surg. Res.*, (accepted, in press).
5. Iwase, K., Takenaka, H., Yagura, A., Ishizaka, T., Ohata, T., Takagaki, M. and Oshima, S. (1992) Hemodynamic alterations during laparoscopic cholecystectomy in patients with heart disease. *Endoscopy*, **24**, 771–773.
6. Richards, W. O., Scovill, W., Shin, B. and Reed, W. (1983) Acute renal failure associated with increased intra-abdominal pressure. *Ann. Surg.*, **197**, 183–187.
7. Kron, I. L., Harman, P. K. and Nolan, S. P. (1984) The measurement of intra-abdominal pressure as a criterion for abdominal re-exploration. *Ann. Surg.*, **199**, 28–30.
8. Kelman, G. R., Swapp, G. H., Smith, I., Benzie, R. J. and Gordon, N. L. M. (1972) Cardiac output and arterial blood-gas tension during laparoscopy. *Br. J. Anesth.*, **44**, 1155–1161.
9. Ivankovichi, A. D., Miletich, D. J., Albrecht, R. F., Heyman, H. J. and Bonnet, R. F. (1975) Cardiovascular effects of intraperitoneal insufflation with carbon dioxide and nitrous oxide in the dog. *Anesthesiology*, **42**, 281–287.
10. Olsen, D. O. (1991) Laparoscopic cholecystectomy. *Am. J. Surg.*, **61**, 339–344.

